# Are Olfactory Cues Involved in Nest Recognition in Two Social Species of Estrildid Finches?

**DOI:** 10.1371/journal.pone.0036615

**Published:** 2012-05-04

**Authors:** E. Tobias Krause, Barbara A. Caspers

**Affiliations:** Department of Animal Behaviour, University of Bielefeld, Bielefeld, Germany; The Australian National University, Australia

## Abstract

Reliably recognizing their own nest provides parents with a necessary skill to invest time and resources efficiently in raising their offspring and thereby maximising their own reproductive success. Studies investigating nest recognition in adult birds have focused mainly on visual cues of the nest or the nest site and acoustic cues of the nestlings. To determine whether adult songbirds also use olfaction for nest recognition, we investigated the use of olfactory nest cues for two estrildid finch species, zebra finches (*Taeniopygia guttata*) and Bengalese finches (*Lonchura striata var. domestica*) during the nestling and fledgling phase of their offspring. We found similar behavioural responses to nest odours in both songbird species. Females preferred the odour of their own nest over a control and avoided the foreign conspecific nest scent over a control during the nestling phase of their offspring, but when given the own odour and the foreign conspecific odour simultaneously we did not find a preference for the own nest odour. Males of both species did not show any preferences at all. The behavioural reaction to any nest odour decreased after fledging of the offspring. Our results show that only females show a behavioural response to olfactory nest cues, indicating that the use of olfactory cues for nest recognition seems to be sex-specific and dependent on the developmental stage of the offspring. Although estrildid finches are known to use visual and acoustic cues for nest recognition, the similar behavioural pattern of both species indicates that at least females gain additional information by olfactory nest cues during the nestling phase of their offspring. Thus olfactory cues might be important in general, even in situations in which visual and acoustic cues are known to be sufficient.

## Introduction

Passeriformes are traditionally regarded as birds which mainly rely on visual and acoustic stimuli. The sense of smell has been neglected due to their relatively small olfactory bulbs [1]. Despite these small olfactory bulbs, studies have recently revealed that songbirds have the capacities to smell in their genetic repertoire [2], [3] and make use of these capacities to avoid predators [4], [5], orientate [6], distinguish between hetero- and conspecifics [7]–[9] and for nest construction [10]–[12]. It has also been demonstrated that fledglings of a colony breeding songbird, the zebra finch (*Taeniopygia guttata*), can find their nest based on olfactory cues [13] and can recognize kin based on olfactory cues [14]. Olfactory identification of the natal nest is adaptive for fledglings, as they have no visual representation of their nest site after leaving the nest for the first time. Thus, in the absence of visual and acoustic cues, olfaction can provide a crucial signal. An interesting question arising from this finding is whether the nest odour also provides a reliable additional signal to parent birds, which could rely on visual and/or acoustic cues alone for nest recognition?

Recognising one's own nest is a crucial skill for successful reproduction. Especially altricial birds should be proficient in finding their own nest as it is necessary to supply care and food to their offspring. This is even more apparent in social-living and colony-breeding species, with high densities of nests within a colony and thus a potentially higher possibility of mismatch. Birds are known to rely on visual cues for nest site recognition [15]–[18], and on acoustic signals from their offspring inside the nest to identify their own nest [19]–[21]. The role of olfaction has been less studied in most avian species, and especially in songbirds. Studies investigating olfactory nest recognition have been focused on birds which cannot rely on visual cues for nest recognition, such as nocturnal birds [22]–[25], nestlings [26] or fledglings, which cannot have a spatial representation of their nest site [13]. Whether the olfactory signature of a nest provides any additional signal used by adult songbirds is, however, less well examined.

To test whether the sense of smell might also be involved in nest recognition in adult social songbirds, we investigated the use of olfactory nest cues in two social estrildid species, zebra finches and Bengalese finches.

We investigated the use of olfaction in nest recognition in adult breeding pairs of both species during the nestling phase of their offspring and after they fledged. At each of the two developmental stages we performed three different odour preference tests with parent birds, giving them the choice between: i) their own nest odour and unused nest material, ii) foreign conspecific nest odour and unused nest material, and iii) own and foreign conspecific nest odour. If olfactory cues are involved in nest recognition, we expect parents to prefer the odour of their own nest over the odour of a foreign conspecific nest.

## Results

### Zebra Finches

#### Odour preference tests at the nestling phase of offspring

**Figure 1:**
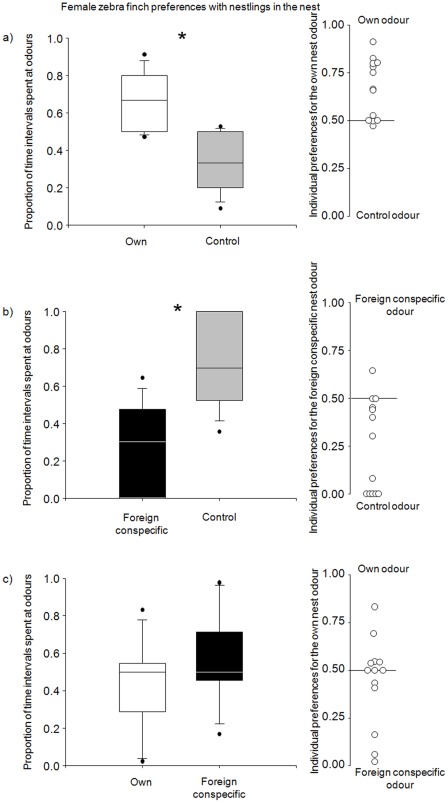
Female zebra finch preferences in three olfactory choice tests. Females were tested in the different test situations (a–c), always having the choice between two different odours. The average (left side) and individual results as dots (right side) of the odour preference test for the adult breeding zebra finch females with 10 day old nestlings in the nest, when tested with a) own nest odour against control odour (Wilcoxon-test, N = 13, Z = −2.667, p = 0.008), b) foreign conspecific nest odour against control odour (Wilcoxon-test, N = 13, Z = −2.606, p = 0.009) and c) own nest odour against foreign conspecific nest odour (Wilcoxon-test, N = 13, Z = −0.866, p = 0.39).

Zebra finch females preferred their own nest odour over a control odour and spent significantly more intervals in the vicinity of their own nest odour (Wilcoxon, N = 13, Z = −2.667, p = 0.008, Fig. 1a). When a foreign conspecific odour and a control odour were presented, zebra finch females avoided the foreign conspecific odour (Wilcoxon, N = 13, Z = −2.604, p = 0.009; Fig. 1b) and spent significantly fewer intervals in the vicinity of the foreign conspecific odour. In the third experiment, where a birds' own nest odour and a foreign conspecific nest odour were presented simultaneously, zebra finch females did not show any preference and spent similar amounts of intervals at both odour stimuli (Wilcoxon, N = 13, Z = −0.866, p = 0.39; Fig. 1c).

We tested 12 zebra finch males (one male died before the tests were conducted). In contrast to females, males in all three test situations showed no preference for and no avoidance of any olfactory stimuli (all Wilcoxon, N = 12, all Z>−0.63, p>0.53; Table 1).

**Table 1:** Results of the odour preference tests of adult breeding males, at the experiments with nestling offspring in the nest, from both species the zebra finch and the Bengalese finch with nestlings in the nest.

	Male's median preferences at nestling phase of offspring in % in the tests		
	Own – Control	Foreign conspecific – Control	Own – Foreign conspecific
Zebra finch males	50–50	47–53	51–49
Bengalese finch males	49–51	44–56	52–48

#### Odour preference tests after fledging of offspring

After the offspring fledged, females did not show any preference or avoidance in any of the three tests (all Wilcoxon, N = 10, all Z>−1.1, p = 0.26; Table 2). Similar to the findings in females, males showed no significant preference or avoidance in the three tests (all Wilcoxon, N = 10, Z>−1.69, p>0.091; Table 2).

**Table 2:** Results of the odour preference tests of zebra finches and Bengalese finches parents after fledging of the offspring.

	Median preferences in % in the tests after fledging of the offspring		
	Own – Control	Foreign conspecific – Control	Own – Foreign conspecific
Zebra Finch males	47–53	42–58	47–53
Zebra Finch females	61–39	52–48	49–51
Bengalese Finch males	65–35	76–34	58–42
Bengalese Finch females	50–50	24–76	50–50

### Bengalese Finches

#### Odour preference tests at nestling phase of offspring

**Figure 2:**
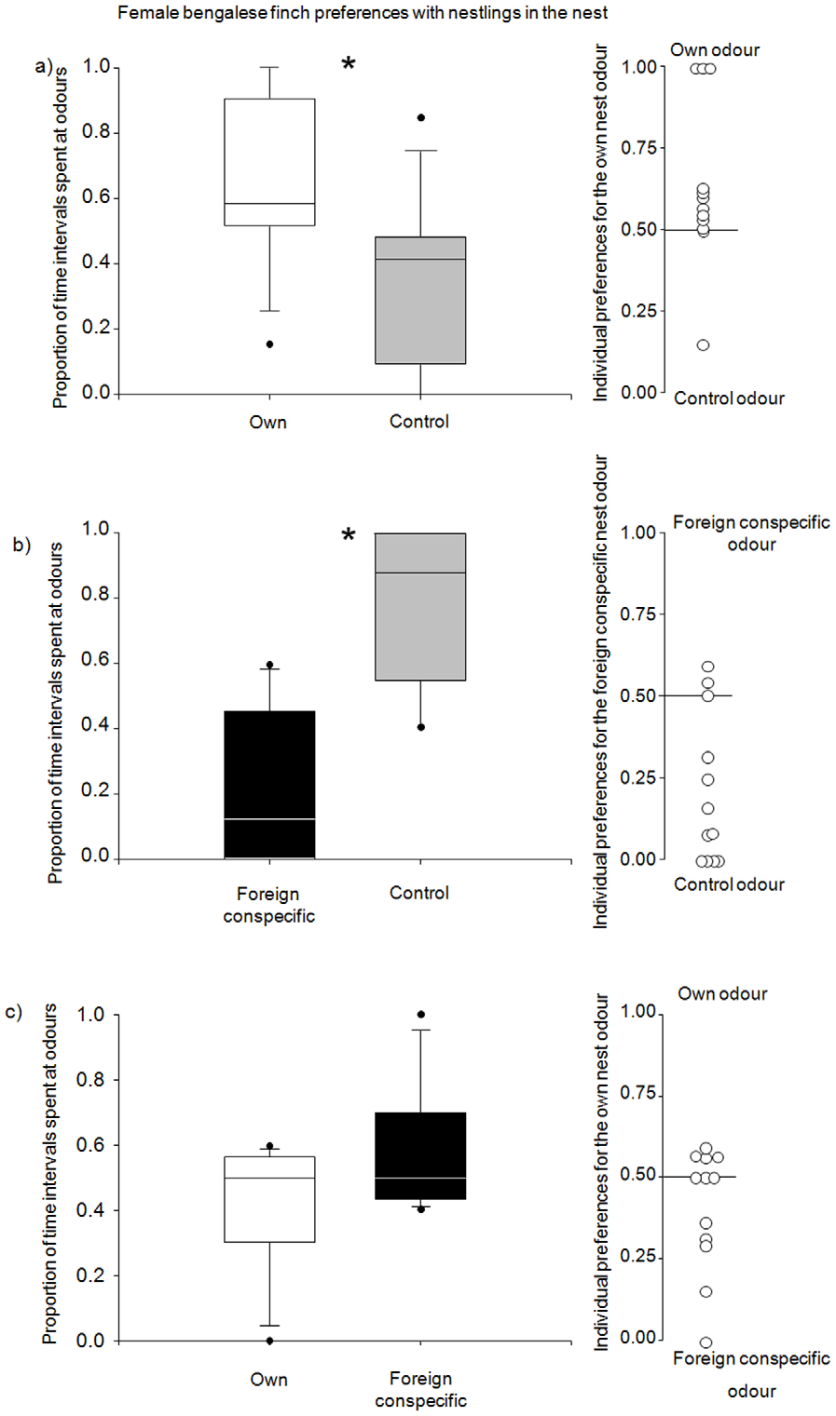
Female Bengalese finch preferences in three olfactory choice tests. Females were tested in the different test situations (a–c), with always having the choice between two different odours. The average (left side) and individual results as dots (right side) of the odour preference test for the adult breeding Bengalese finch females with 12 day old nestlings in the nest, when tested with a) own nest odour against control odour (Wilcoxon-test, N = 12, Z = −2.227; p = 0.026), b) foreign conspecific nest odour against control odour (Wilcoxon-test, N = 12, Z = −2.681, p = 0.007) and c) own nest odour against foreign conspecific nest odour (Wilcoxon-test, N = 12, Z = 1.481, p = 0.139).

Bengalese finch females preferred their own nest odour compared to a control odour and spent significantly more time in the vicinity of their own nest odour (Wilcoxon, N = 12, Z = −2.227, p = 0.026, Fig. 2a). When a foreign conspecific odour and a control odour were presented, Bengalese finch females avoided the foreign conspecific odour (Wilcoxon, N = 12, Z = −2.681, p = 0.007; Fig. 2b) and spent significantly less time in the vicinity of the foreign conspecific odour. In the third experiment, where a birds' own nest odour and a foreign conspecific odour were presented simultaneously, Bengalese finch females did not show any preference and spent similar amounts of time at both odour stimuli (Wilcoxon, N = 12, Z = −1.481, p = 0.139; Fig. 2c).

In contrast to females, Bengalese finch males in all three test situations showed no preference for and no avoidance of any olfactory stimuli (all Wilcoxon, N = 12, all Z>−0.82, p>0.41; Table 1).

#### Odour preference tests after fledging of offspring

After the offspring fledged, Bengalese finch females did not show any preference or avoidance in any of the three tests (all Wilcoxon, N = 12, all Z>−1.75, p>0.08; Table 2). Similar to the findings in females, Bengalese finch males showed no significant preference or avoidance in the three tests (all Wilcoxon, N = 12, Z>−1.50, p>0.13; Table 2).

## Discussion

Our results show that adult females of two social estrildid finches were able to perceive nest odours and show specific behavioural responses depending on the olfactory stimulus (own nest odour; foreign conspecific nest odour) and that their behaviour depended on the developmental stage of their offspring. During the nestling phase of their offspring, females of both species preferred their own nest odour over a control and avoided a foreign conspecific nest odour against a control, but we did not find a preference for the own nest odour, when given the own nest odour and the foreign conspecific nest odour simultaneously. Males, in contrast, did not show any behavioural preference at all. The fact that the behavioural responses of both species are very similar leads to the conclusion that females may gain additional information provided by olfactory nest cues.

Contrary to our expectations, we did not find a preference, consistently in the females of both species, for the own nest odour, when given the own odour and the foreign conspecific odour simultaneously. Maternal olfactory nest recognition seems to be context-dependent, i.e., the bird's own nest odour is preferred over a control, and foreign conspecific nest odour is avoided compared with a control. Since the first two experiments clearly show that females are able to perceive the odours of the own nest and that of the foreign conspecific nest, we can rule out the possibility that the lack of preference is due to a general lack of scent perception. However, it might be possible that when presented simultaneously, both olfactory stimuli are difficult to differentiate for adult birds, different to the ability and reactivity of fledglings in this simultaneous choice situation [13], [14]. Another explanation might be that the usage of olfactory nest cues is neglected in adult birds when being faced directly with their own and a foreign odour, as this represents a situation in which the birds are directly in front of two adjacent nests. In such a situation birds may rely more on visual and acoustic cues than on olfactory cues to avoid errors in nest recognition [15]–[21].

A lack of preference, when presented with two nest odours simultaneously, has also been found in some petrels. Leach storm petrel (*Oceanodroma leucorhoa*) chicks, for example, show a clear preference for their nest odour, when given the choice between their own nest odour and other colony material. However, when given the choice between their own nest odour and a conspecific nest odour, the preference was less pronounced [25]. The same effect was found in European storm petrel (*Hydrobates pelagicus*) chicks [27]. When given two odour stimuli simultaneously, more than half of the test individuals did not show a preference at all. Both studies argued that the discrimination between two similar types of odours is more difficult. These parallels in the results between petrels and estrildid finches, despite differences in life-history and ecology, seems to indicate that the general ability to recognise odours is also dependent on the specific contexts.

It might be beneficial for female zebra finches and female Bengalese finches to prefer the odour of their own nests, and to avoid the odour of the foreign conspecific nest during the nestling phase of their offspring. The nestling phase is the most important, most time-consuming and energetically most costly phase for parents, especially in altricial birds [28], [29]. Offspring growth is highest during the nestling phase, and nutritional stress during this early phase can have long-lasting fitness consequences [30]–[35]. Thus, the importance of identifying the own nest is high, and so are the costs of mismatch, e.g., feeding other than one's own chicks [28]. Additionally, it seems likely that aggression towards nest intruders is higher during the nestling phase than afterwards because the parental costs are highest during this phase [36]. Females avoiding foreign conspecific nests might benefit from doing so by avoiding conflicts and potential injuries or time loss.

It is also very likely that adult birds do not only recognize their nest based on one single sensory mode, but more likely on multi-modal cues. Experimental evidence suggests that each single cue can be sufficient for nest recognition (visual cues [17]–[18], acoustic cues [19] and olfactory cues [13], [22], [24]), but might probably be replaced by others when necessary. Single sensory modes are error-prone, which might have facilitated the evolution of multi-modal nest recognition. For example, in a noisy and/or crowded environment such as a large colony, acoustic cues might be error-prone for nest-recognition [37]–[39]. The same is true for visual cues at dusk or dawn and/or in dense bushes, since light conditions might be poor and thus visual perception is more likely to be error-prone [40]. In such cases where visual and/or acoustic cues are insufficient for nest recognition, olfactory cues might provide additional information.

Even though both parents participate in parental care, in both study species males did neither show a preference for their own nest nor avoided their foreign conspecific nest odour. This might be due to the fact that adult males are not capable of olfaction. However, this explanation seems very unlikely since there was no sex specific difference in odour preference in zebra finch fledglings [13], [14]. Another explanation might be that males do not rely on olfactory cues and either use visual cues for nest recognition [15]–[17] or use acoustic cues for offspring recognition [19]. It might also be possible that males in general do not discriminate between cues of their own and a foreign brood [41]–[43]. Extra-pair paternity is rare in zebra finches [44]. Hence, the selection pressure for the evolution of a nest recognition mechanism in males, which is very likely to be linked with offspring recognition in altricial birds, might be lower in zebra finches and Bengalese finches compared with other songbird species.

The females' preference for their own nest odour and the avoidance of a foreign conspecific nest odour decreases after offspring fledged. After fledging, juveniles are occasionally fed by the parents outside the nest [36], [45]. This might decrease the need to distinguish between different nests and their respective odours. The difference in odour preference subject to age differences of the chicks might also be due to females changing their behavioural reaction due to hormonal changes after fledging of the offspring, as has been shown in mice [46], [47].

The similar pattern, which we found in two social songbirds of the same family (*Estrildidae*), raises the question whether the ability to use olfactory cues as another reliable sense for nest recognition, might have evolved as an adaptation to group-living and colony-breeding or whether olfactory nest cues might encode additional information about the offspring inside the nest. In altricial birds the nest is directly linked with the offspring inside, thus olfactory nest cues used by adult females might also provide information about the offspring inside the nest [14]. Whether female zebra finches, or female Bengalese finches use olfactory cues to identify their own offspring needs to be tested in future studies. However, only recently some studies have been focused on olfactory kin recognition in birds [14], [48], [49].

In conclusion, we demonstrated that adult breeding females of two estrildid finch species, zebra finch (*Taeniopygia guttata*) and Bengalese finch (*Lonchura striata var. domestica)*, show a similar pattern in their use of olfactory cues for nest recognition. This similar pattern makes it difficult to escape the conclusion that olfaction is of importance for adult songbirds in nest recognition and probably further contexts.

## Methods

### Ethics Statement

The experiments were carried out according to the German laws for experimentation with animals. No additional licences were required for performing non-invasive experiments with birds. Breeding and housing of the birds was conducted under permission of the Veterinäramt Bielefeld, Germany (# 530.421630-1, 18.04.2002) according to the German Tierschutzgesetz §11. After the study all birds remained in the laboratory stock at the University of Bielefeld.

### Breeding conditions

We carried out the breeding for the zebra finches from August 2009 until February 2010 and for the Bengalese finches from July 2010 until June 2011 at the University of Bielefeld, Germany. In each of the two breeding attempts we allowed randomly assigned pairs to breed in three compartment cages (115×40×30 cm) with a wooden nest box (15×15×15 cm) attached to the central compartment. We used 26 adult zebra finches (13 males, 13 females) and 24 adult Bengalese finches (12 females, 12 males) in the experiments. These thirteen pairs of zebra finches (*Taeniopygia guttata*) and twelve pairs of Bengalese finches (*Lonchura striata var. domestica*) bred and successfully reared chicks (average brood size during nestling phase; ZF: 2.5 chicks ±1.2 SD; BF: 3.2 chicks ±1.6 SD). Food and water were provided *ad libitum* at both sides of the cages to ensure that the birds did not develop a side preference based on the location of the food source. Coconut fibres were provided on the floor of the central compartment as nest material. Nest boxes were checked daily to record hatching dates of chicks.

In the wild, zebra finches (*Taeniopygia guttata*) breed in colonies with up to 50 pairs [36], [50]. The density of their colonies varies from one breeding pair in each bush to more than a dozen pairs, dependent on the specific local ecological situation [36], [51]. Bengalese finches (*Lonchura striata var. domestica*), are regarded as the domesticated form of the white-rumped munia (*Lonchura striata*), another species of the monophyletic group of the *Estrildidae* [52]. Bengalese finches are group-living birds [45], [53], [54] which are very unaggressive to conspecifics during breeding [55]. Zebra finches are also less aggressive during breeding; they only show territorial behaviour with regard to the nest itself [36]. Zebra finches usually lay clutches of 4–6 eggs, which are incubated for 11–14 days until hatching of the chicks [36]. Bengalese finches usually lay clutches of 5–6 eggs and the incubation period lasts for 15–19 days [45], [55]. In both species males and females participate in nest construction, incubation and parental care [36], [56].

### Experimental procedures

All parent birds were tested during the two major developmental stages of the offspring. First, during the nestling phase of their chicks (ZF at a median brood age of 10 days; BF at a median brood age of 12 days) and the second set of experiments was conducted shortly after the offspring fledged (ZF at a median brood age of 23 days; BF at a median brood age of 26 days). Zebra finches usually fledge at around day 19 [36], [53], [57] and Bengalese finches usually at around day 24 [45], [55]. The experiments were conducted in the home cages. During experiments, the other parent, the natal nest box, and the offspring were removed from the home cage. Instead of the natal nest box, two artificial test nest boxes were attached to the two side compartments of the cage. The test nest boxes were filled with fresh coco fibres shaped to resemble a nest. In the back wall of the test nest boxes, a round hole (diameter 7.5 cm) was present, covered by a wire mesh basket in which odour samples were placed. A fan was placed behind the basket to circulate air through the odour sample into the test nest box [13], [14]. To obtain the odour samples, we removed nest material (approximately 2.5 g) from the home nest and from a foreign conspecific nest, thus the foreign nest odour was still from the same species, i.e. zebra finches received the foreign odour from another unfamiliar foreign zebra finch nest and Bengalese finches received the foreign odour from another unfamiliar foreign Bengalese finch nest. Foreign conspecific nests were randomly chosen from pairs with offspring of similar age. The nest material used was partly covered with faeces. Odour samples were placed in pouches of synthetic gauze and placed into the mesh basket behind the artificial nest box, making the sample invisible to test animals.

We used each nest of a breeding pair as a foreign conspecific stimulus for only one other pair and the same breeding pair provided the foreign conspecific nest odour stimulus for the two testing periods. Prior to each test, we placed the odour samples in the baskets and turned on the fans for 20 minutes to allow the odour stimuli to evaporate into the test cage. Afterwards, individuals were tested for five minutes. To control for side preferences, odour samples were exchanged, and we turned on the fans for another 20 minutes for odour evaporation. The same individual was then tested for another five minutes. Thus, each individual was tested in total for 10 min in each of the three experiments. The starting sides for the odour samples and test sequences were randomised. In the 20-minute evaporation intervals before and between the two test phases of each experiment, opaque slides were placed between the central part and the side compartments to prevent test individuals from moving into the side compartments.

During the tests, we recorded the location of the individual every three seconds. We counted the time intervals spent by each individual in one of the test nest boxes or on the perch directly in front of the nest. The use of all other places and perches was not considered to be choice [13], [14]. In the experiments at day 10 zebra finch females spent on average 82.2 intervals ±10.1 SD, and zebra finch males on average 112.4 intervals ±10.7 SD, of the 200 possible intervals in the choice areas. In the experiments at day 23 zebra finch females spent on average 82.0 intervals ±5.7 SD, and zebra finch males on average 91.0 intervals ±17.9 SD in the choice areas. In the experiments with the Bengalese finches we additionally noted whether the individual changed the location within the last three second time interval. Afterwards we measured the times as follows. If the location was changed within a 3-second interval the time was scored as 1.5 s; otherwise, it was scored as 3 seconds (after [58]). In the experiments at day 12 Bengalese finch females spent on average 323.5 seconds ±66.7 SD, and Bengalese finch males on average 306.1 seconds ±18.0 SD, of the 600 possible seconds in the choice areas. In the experiments at day 26 Bengalese finch females spent on average 170.9 seconds ±27.2 SD, and Bengalese finch males on average 211.1 seconds ±23.9 SD in the choice areas.

For all subjects we counted the intervals or time the subject was sitting in the choice areas (in the test nest boxes or on the perch directly in front of the nest). For statistical analysis we calculated the respective percentage of choice measured by the number of intervals an individual spent in the respective preference zone divided by the number of intervals an individual spent in both preference zones.

In each experimental session (at nestling and fledgling phase of the offspring), three tests were conducted for the parents of each species, each with two different olfactory stimuli simultaneously presented. We performed the three tests in randomised order: i) odour of the birds' own nest (some nest material and faeces taken from the own nest) against control odour (unused coco fibres); ii) foreign conspecific nest odour (nest material and faeces taken from a foreign conspecific, with same-aged chicks) against control odour; and iii) odour of the birds' own nest against foreign conspecific nest odour.

### Statistical analysis

To test for odour preferences, we compared the percentage of choice adult males and females spent in proximity (choice areas) with the stimulus odours using a Wilcoxon Signed Ranks test. All statistical analyses were performed using SPSS 18.0. All tests were two-sided, and the significance level was set to α = 0.05.
